# Supplementary material for: O-GlcNAcylation is required for B cell homeostasis and antibody responses
Source: Nat Commun. 2017 Nov 30;8:1854. doi: 10.1038/s41467-017-01677-z (PMC5707376; doi:10.1038/s41467-017-01677-z)
Supplement: Supplementary file 1 — Supplementary Information [file 41467_2017_1677_MOESM1_ESM.pdf]

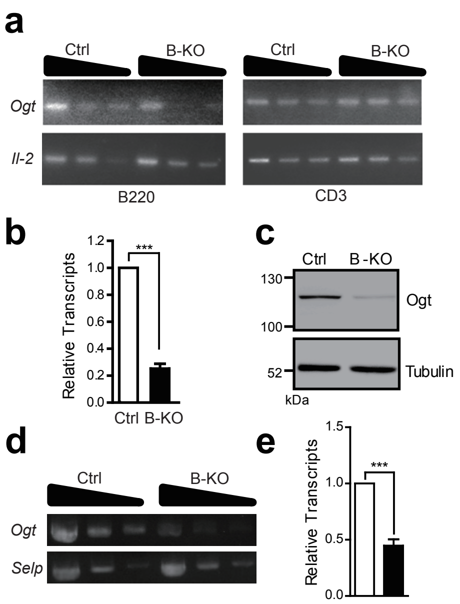

Supplementary Figure 1. B-KO mice demonstrate deletion of *Ogt* in B cells.

a−c. Genomic DNA (a), mRNA (b) and protein lysates (c) were prepared from splenic B cells of Ctrl and B-KO mice. The deletion efficiency of *Ogt* allele was determined by semi-quantitative PCR (a), RT-qPCR (b) and immunoblotting (c), respectively.

d, e. Genomic DNA isolated from bone marrow B220^{+} B cells was subjected to semi-quantitative PCR (d) and RT-qPCR analysis (e) examining the levels of *Ogt* (d) and Ogt mRNA (e) in Ctrl and B-KO bone marrow.

Results in (b) and (e) are the mean ± s.e.m. (n = 3). *** $P < 0.001$ (two-tailed unpaired *t*-test). Three-fold serial dilutions of genomic DNA were used in (a) and (d). *Il-2* and P-selectin (*Selp*) were used as the internal control in (a) and (d), respectively. β-actin mRNA was used to normalize Ogt mRNA in (b) and (e). Tubulin is the protein loading control in (c)

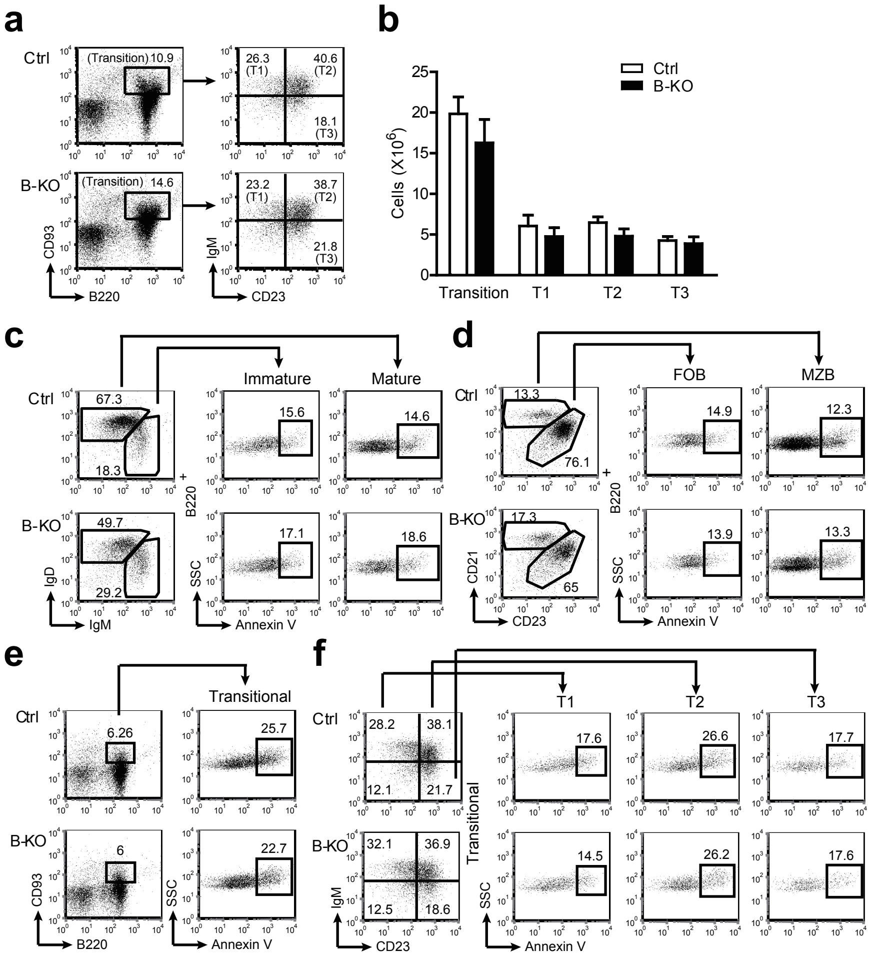

Supplementary Figure 2. More mature B cells in B-KO mice are apoptotic

a. Dot plots showing the populations of various B cell subsets in spleen of Ctrl and B-KO mice. The numbers shown in dot plots are the percentages in quadrants or gates.

b. The bar graphs showing the number of various B cell subsets (Transitional, T1, T2 and T3) in spleen of Ctrl and B-KO mice. Data are mean ± s.e.m. (n = 3) (two-tailed unpaired t-test).

c. Frequency of apoptotic (Annexin V^+) splenic immature and mature B cells in Ctrl and B-KO mice.

d. Frequency of apoptotic marginal zone (MZ) and follicular (FO) B cells in spleen of Ctrl and B-KO mice.

e, f. Frequency of apoptotic transitional B cells (f) as well as T1, T2 and T3 (g) in spleen of Ctrl and B-KO mice.

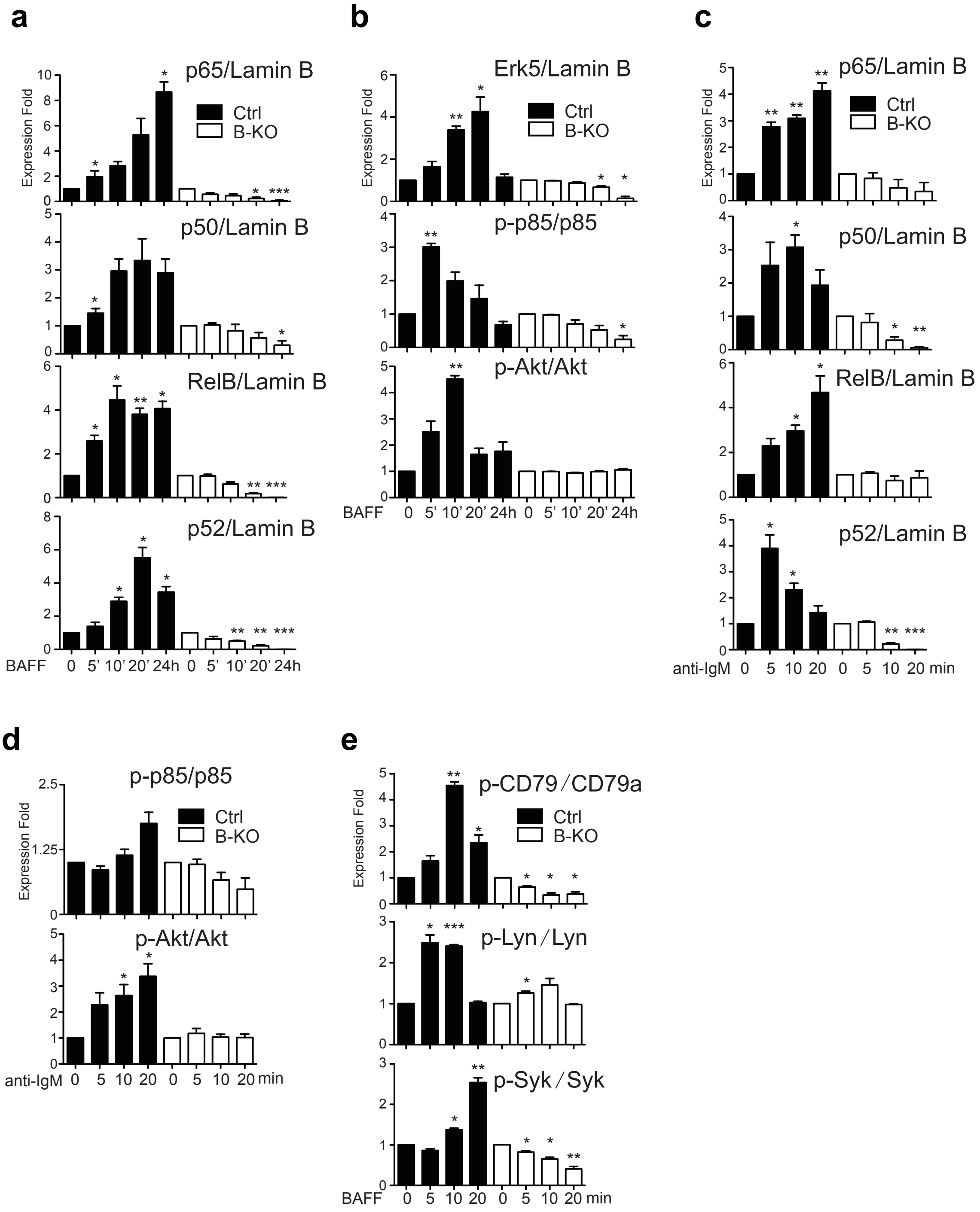

Supplementary Figure 3. Quantification and statistical analyses of Figure 2e, 2f, 3c, 3d and 3g

Quantification results of immunoblotting from Figure 2e, 2f, 3c, 3d, and 3g are show in (a), (b), (c), (d) and (e), respectively. The relative fold expression of the indicated protein or phosphorylated protein was calculated based on normalization of the band intensity to the band intensity of the internal control (β-actin or lamin B) or its corresponding non-phosphorylated counterpart, and then compared with the ratio of the non-stimulated group. Data are mean ± s.e.m. (n = 3). * $P < 0.05$, ** $P < 0.01$, *** $P < 0.001$ (two-tailed unpaired t-test).

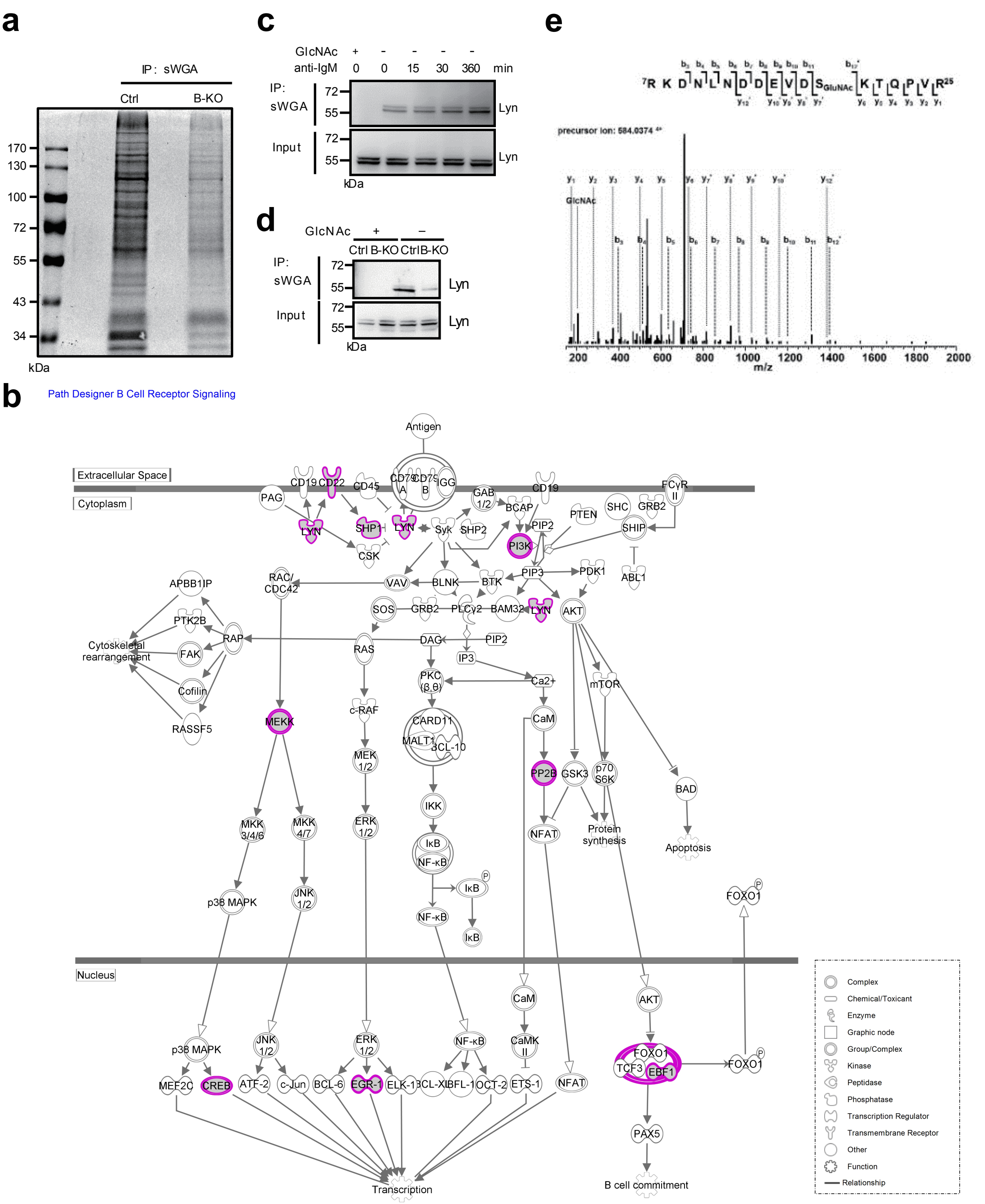

Supplementary Figure 4. Identification of *O*-GlcNAcylated proteins in B cells

a. SDS-PAGE stained with Instant Blue™ (Expedeon, San Diego, CA, USA) showing the protein bands from the lysates of Ctrl and B-KO splenic B cells, pulled down by sWGA.

b. IPA showing the BCR signalling pathway associated proteins identified by sWGA pull-down coupled with mass spectrometric analysis. The identified proteins are circled in red.

c. Immunoblot (IB) showing the levels of Lyn pulled-down by sWGA from the lysates of C57BL/6 splenic B cells stimulated with anti-IgM (10 μg ml^{-1}) at various time points. GlcNAc (0.5 M) was also added to the lysates from un-stimulated B cells in sWGA pull-down.

d. IB showing the level of Lyn that was pulled-down by sWGA from the lysates of Ctrl and B-KO splenic B cells. GlcNAc (0.5 M) was also added to the lysates in the indicated sWGA pull-down.

e. Mapping of the *O*-GlcNAc site at S19 of mouse Lyn using HCD fragmentation. Signal at m/z 204.0853 indicates the presence of GlcNAc modification. The fragmented ion labels with an asterisk (*) represent the neutral loss of GlcNAc.

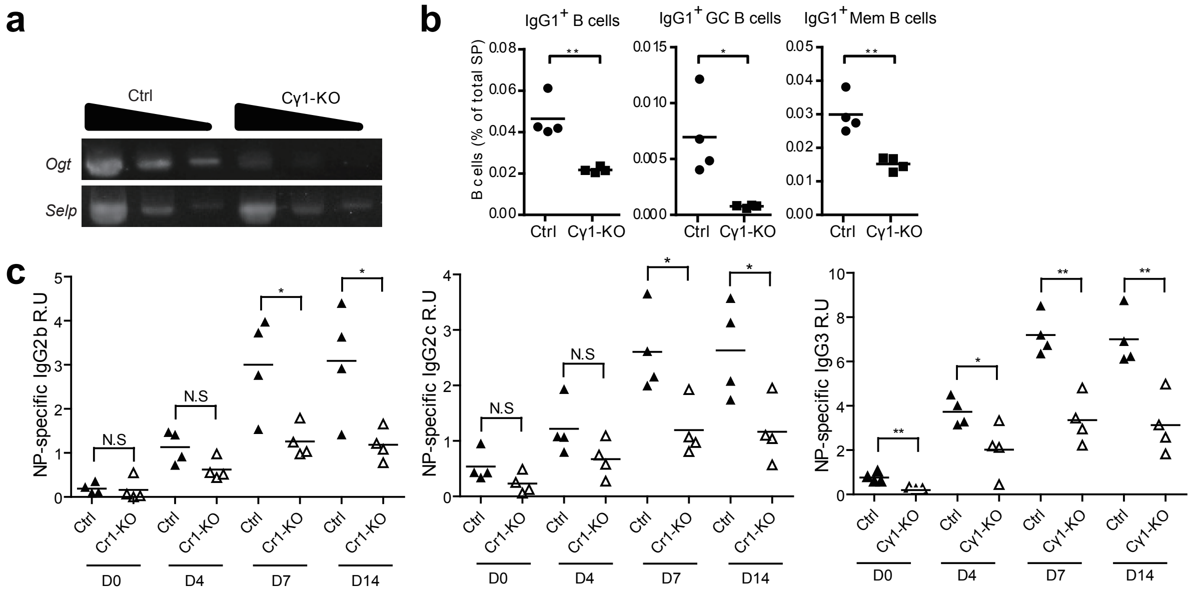

Supplementary Figure 5. Protein *O*-GlcNAcylation is important for mounting GC and antibody responses

a. IgG1+ B cells were sorted from Ctrl and Cγ1-KO splenic B cells that were stimulated with anti-IgM (25 μg ml^{-1}), anti-mouse CD40 (1 μg ml^{-1}) and IL-21 (200 ng ml^{-1}) for 2 days. Semi-quantitative genomic PCR was used to examine the deletion of *Ogt* in sorted class-switched B cells. Three-fold serial dilutions of genomic DNA were used. P-selectin (*Selp*) was used as the internal control.

b. The percentage of IgG1+ GC B cells (IgG1+ GL7+ $CD38^{lo}$) and IgG1+ memory B cells (IgG1+ GL7− $CD38^{hi}$) in total splenocytes 14 days after NP-KLH immunization. Data are the mean ± s.e.m. (n = 4). * $P < 0.05$, ** $P < 0.01$ (two-tailed unpaired t-test).

c. NP-specific IgG2b, IgG2c and IgG3 levels in sera of Ctrl and Cγ1-KO mice after secondary NP-KLH immunization at the indicated days were measured by ELISA. Data are the mean ± s.e.m. (n = 4). N.S., not significant, * $P < 0.05$, ** $P < 0.01$ (two-tailed unpaired t-test).

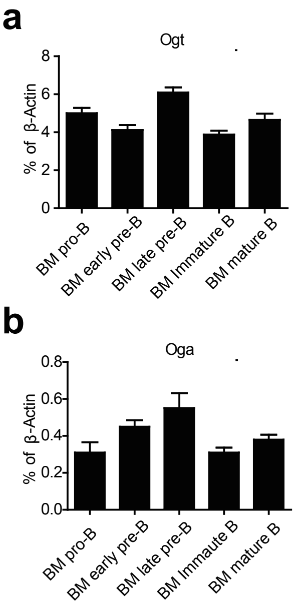

Supplementary Figure 6. The mRNA levels of Ogt and Oga in B cell development

RT-qPCR showing the mRNA levels of Ogt and Oga in various stages of B cells in bone marrow of C57BL/6 mice. Results are mean ± s.e.m. (n = 3) (two-tailed unpaired t-test).

Fig. 1a

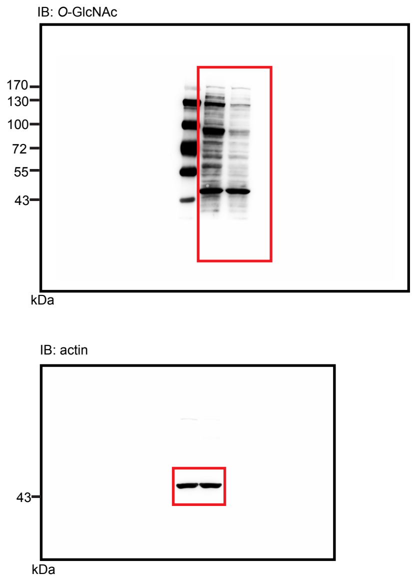

Supplementary Figure 7. Uncropped scanned images of immunoblots from Figure 1

Fig. 2e

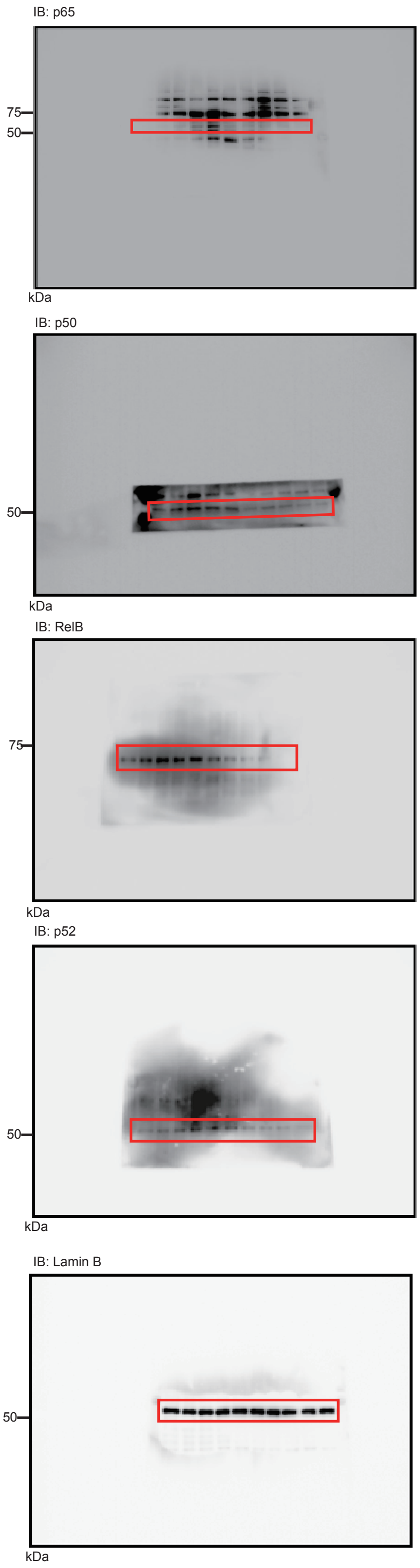

Fig. 2f

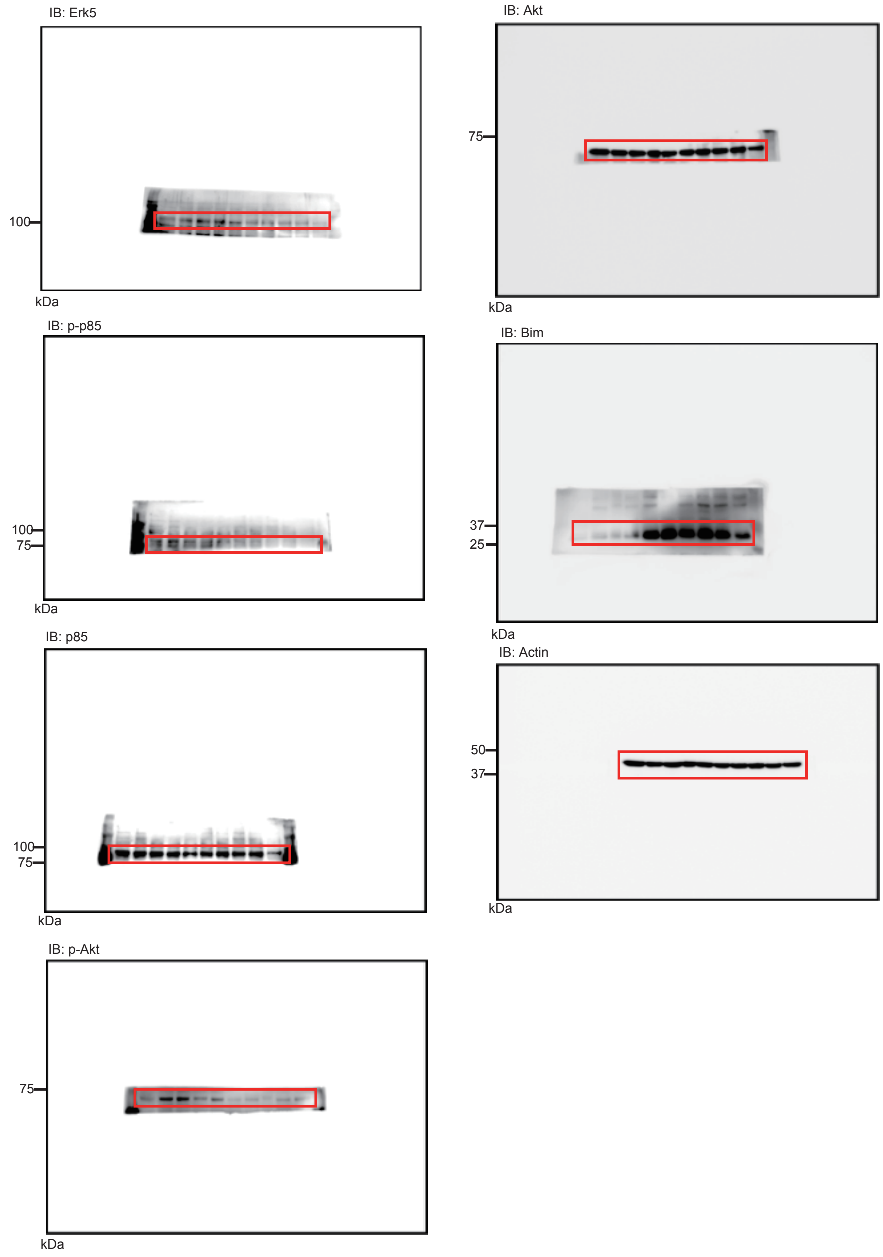

Supplementary Figure 8. Uncropped scanned images of immunoblots from Figure 2

Fig. 3b

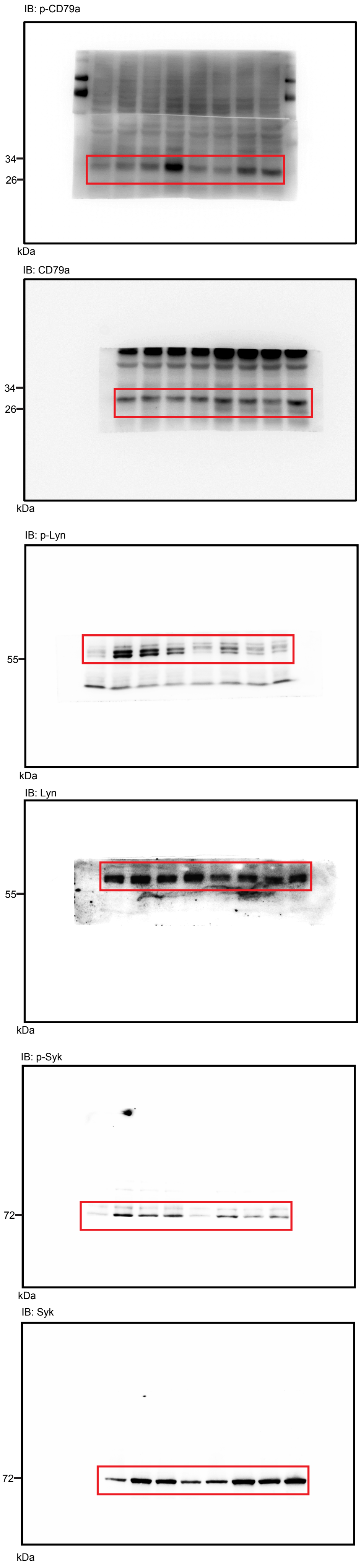
IB: p-CD79a
34
26
kDa
IB: CD79a
34
26
kDa
IB: p-Lyn
55
kDa
IB: Lyn
55
kDa
IB: p-Syk
72
kDa
IB: Syk
72
kDa

Fig. 3c

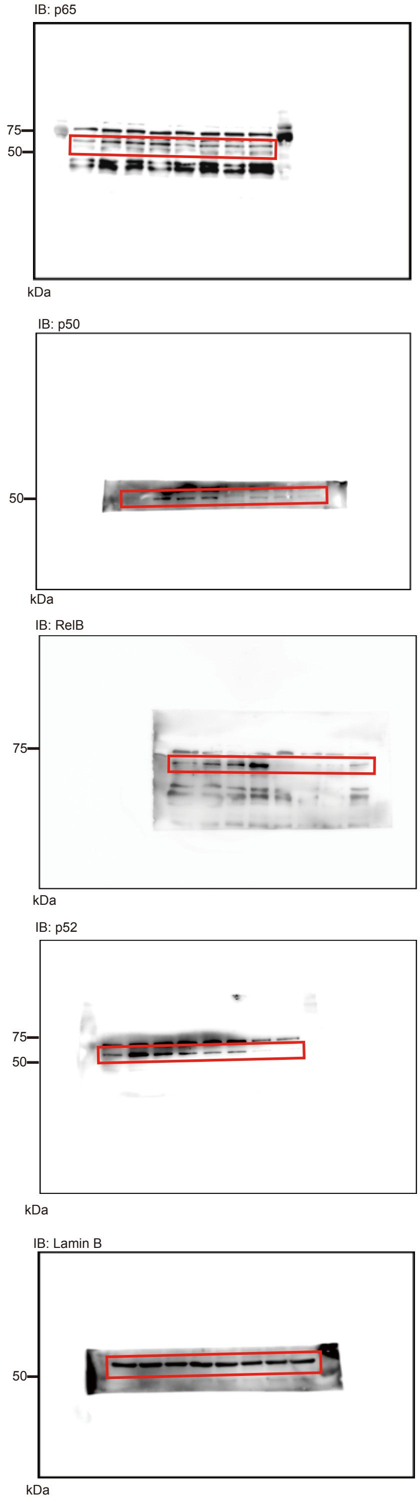
IB: p65
75
50
kDa
IB: p50
50
kDa
IB: RelB
75
kDa
IB: p52
75
50
kDa
IB: Lamin B
50
kDa

Fig. 3d

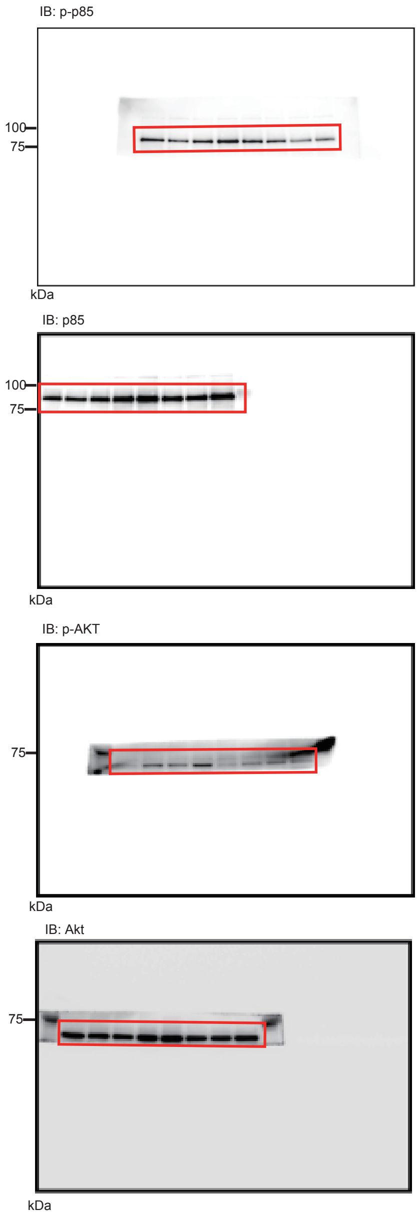

Fig. 3g

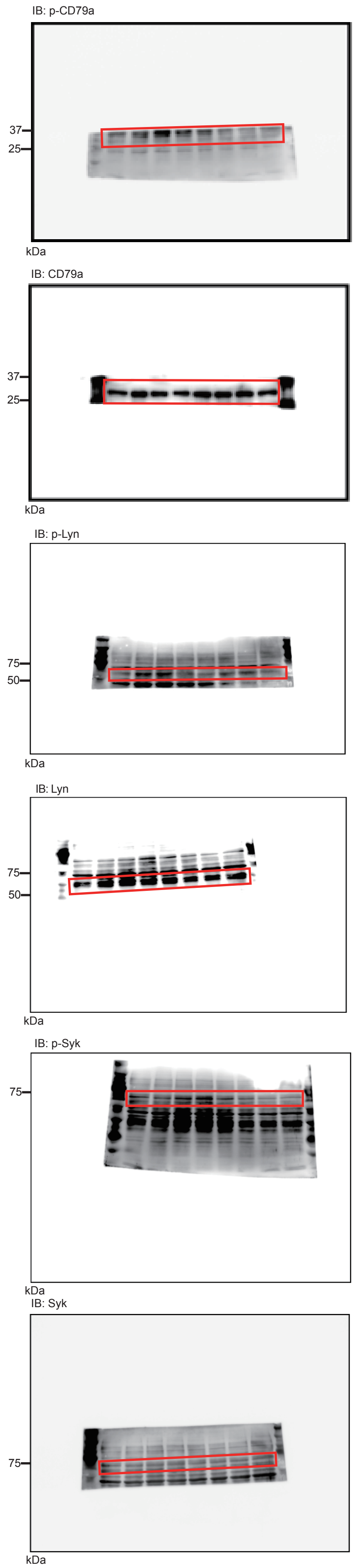

Supplementary Figure 9. Uncropped scanned images of immunoblots from Figure 3

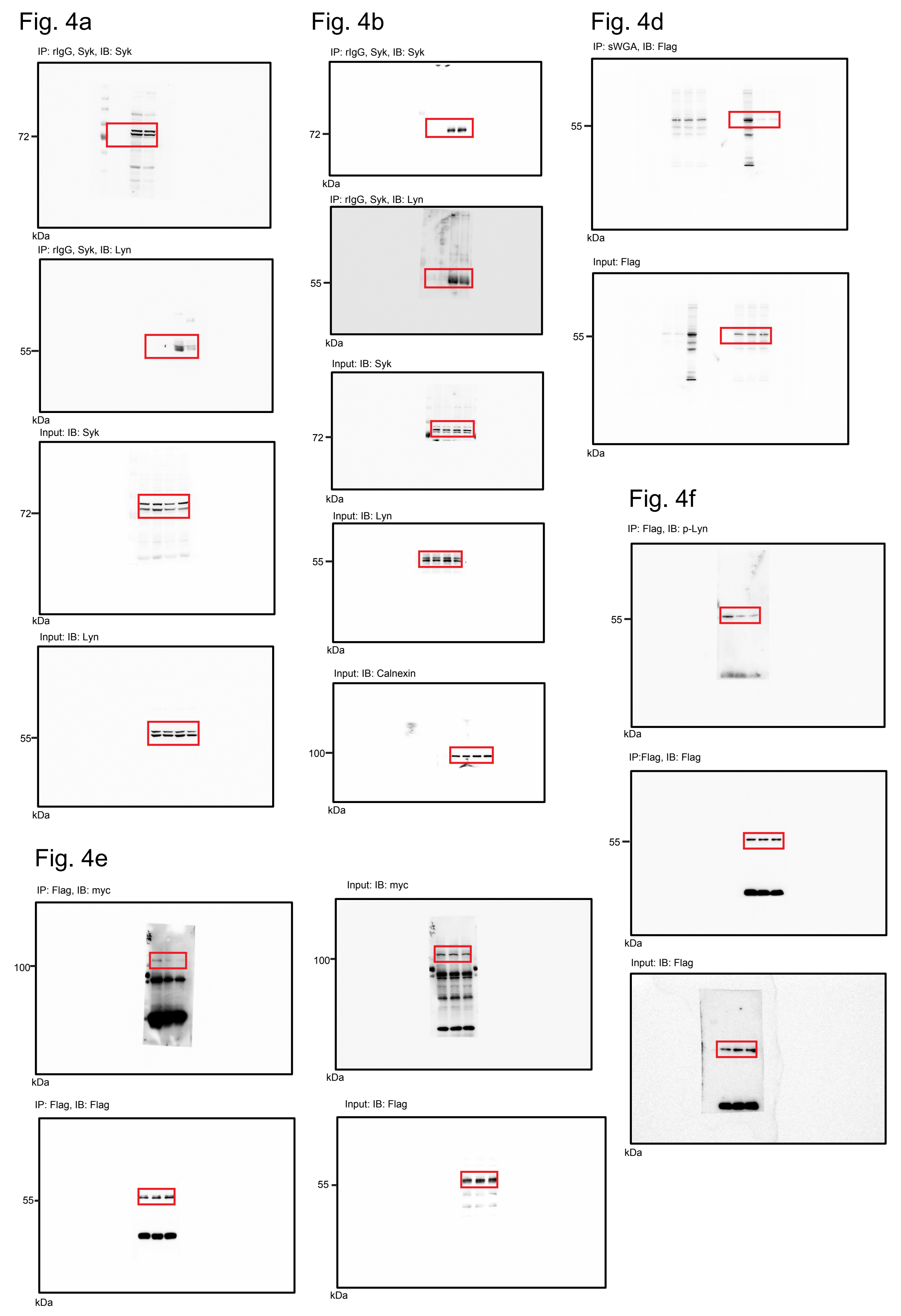

Supplementary Figure 10. Uncropped scanned images of immunoblots from Figure 4

Sup. Fig. 1a

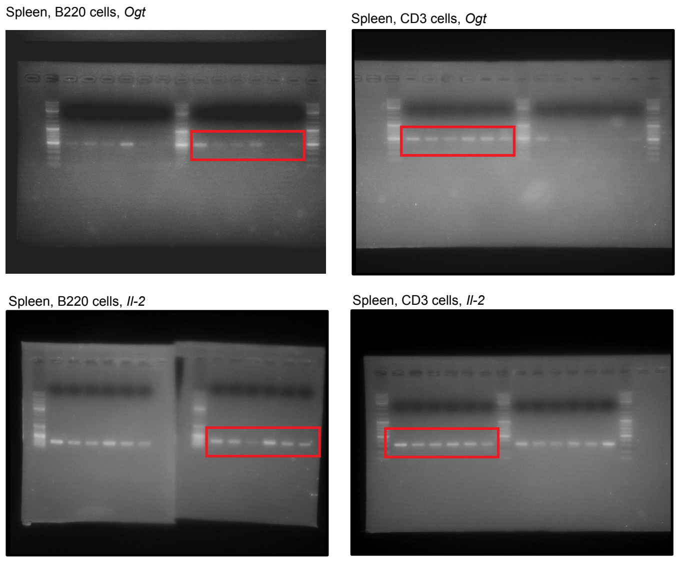

Sup. Fig. 4c

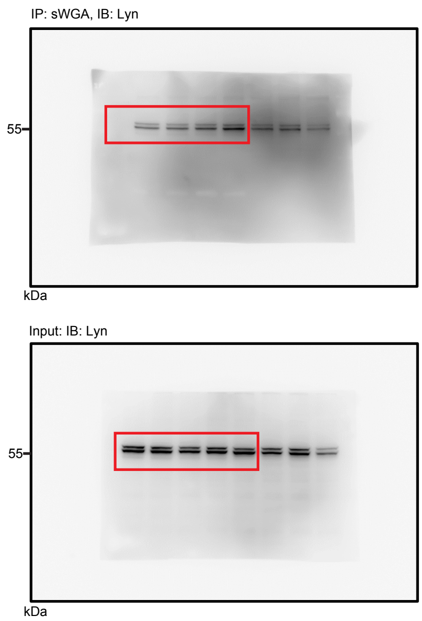

Sup. Fig. 1c

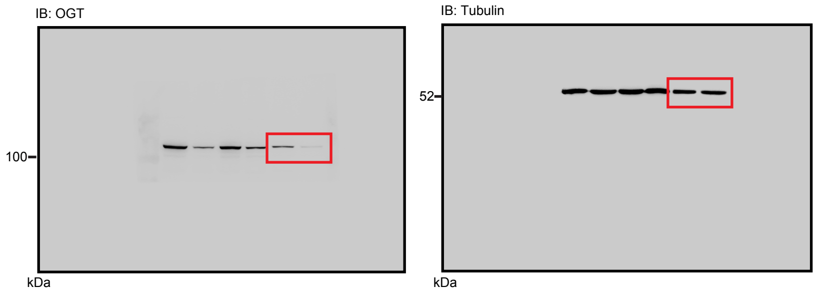

Sup. Fig. 4d

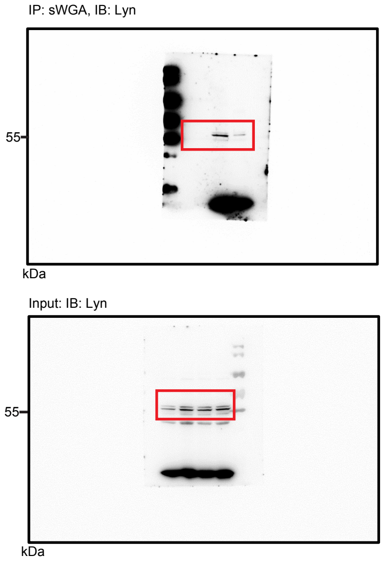

Sup. Fig. 1d

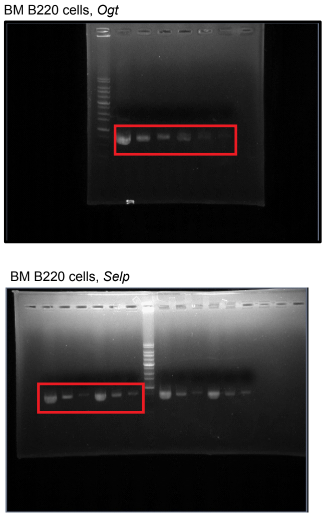

Sup. Fig. 4a

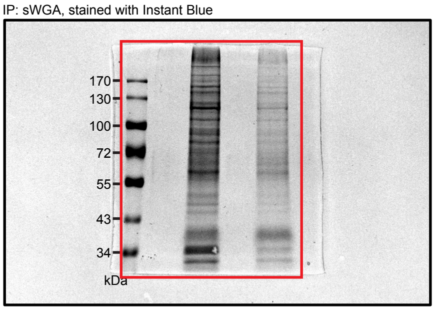

Sup. Fig. 5a

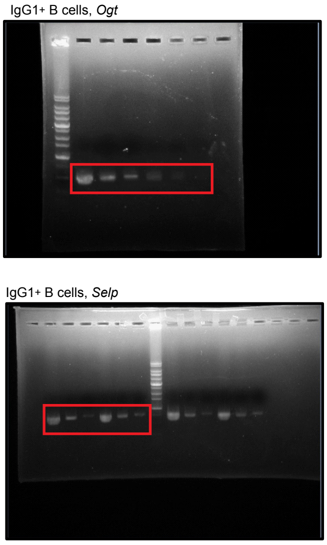

Supplementary Figure 11. Uncropped scanned images of agarose gels, polyacrylamide gel and immunoblots from Supplementary Figures

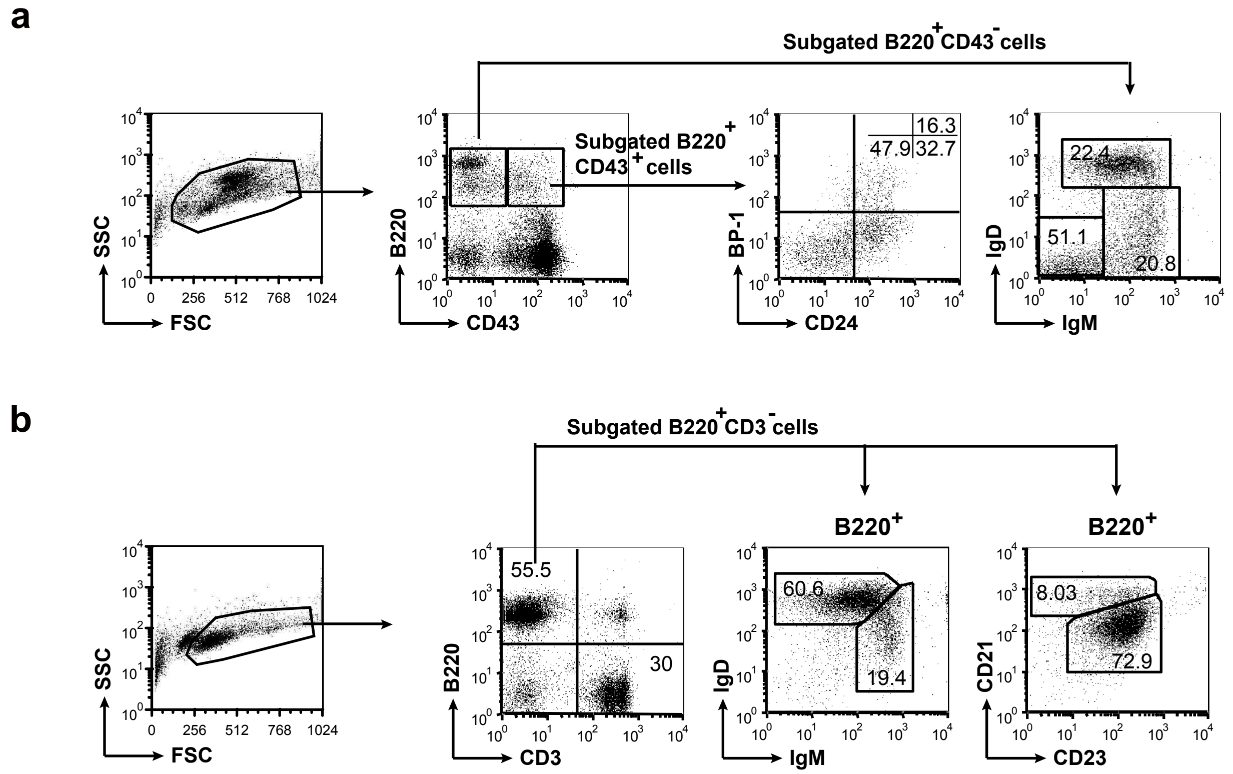

Supplementary Figure 12. Gating strategy for Figure 1

a. Gating strategy for Figure 1c. Bone marrow cells were first gated by size and granularity (FSC and SSC), and then by B220 and CD43 expression (B220$^+$CD43$^+$: GL pro-B, pro-B and early pre-B cells; B220$^+$CD43$^-$: late pre-B, immature B and mature B cells). B220$^+$CD43$^+$ cells were further gated by CD24 and BP-1 expression for GL pro-B (CD24$^-$BP-1$^-$), pro-B (CD24$^+$BP-1$^-$), and early pre-B (CD24$^+$BP-1$^+$) cells. B220$^+$CD43$^-$ cells were further gated by IgM and IgD expression for late pre-B (IgM$^-$IgD$^-$), immature B (IgM^{hi}IgD$^{lo\text{-}int}$) and mature B (IgM$^{int\text{-}hi}$IgD^{hi}) cells.

b. Gating strategy for Figure 1e. Splenic cells were first gated by size and granularity (FSC and SSC), and then by B220 and CD3 expression for B cells (B220$^+$CD3$^-$). B220$^+$CD3$^-$ B cells were further gated by IgM and IgD expression for immature (IgM^{hi}IgD^{lo}) or mature (IgM^{lo}IgD^{hi}) B cells, or by CD23 and CD21 expression for MZB (CD21^{hi}CD23^{lo}) or FOB (CD21^{lo}CD23^{hi}) cells.

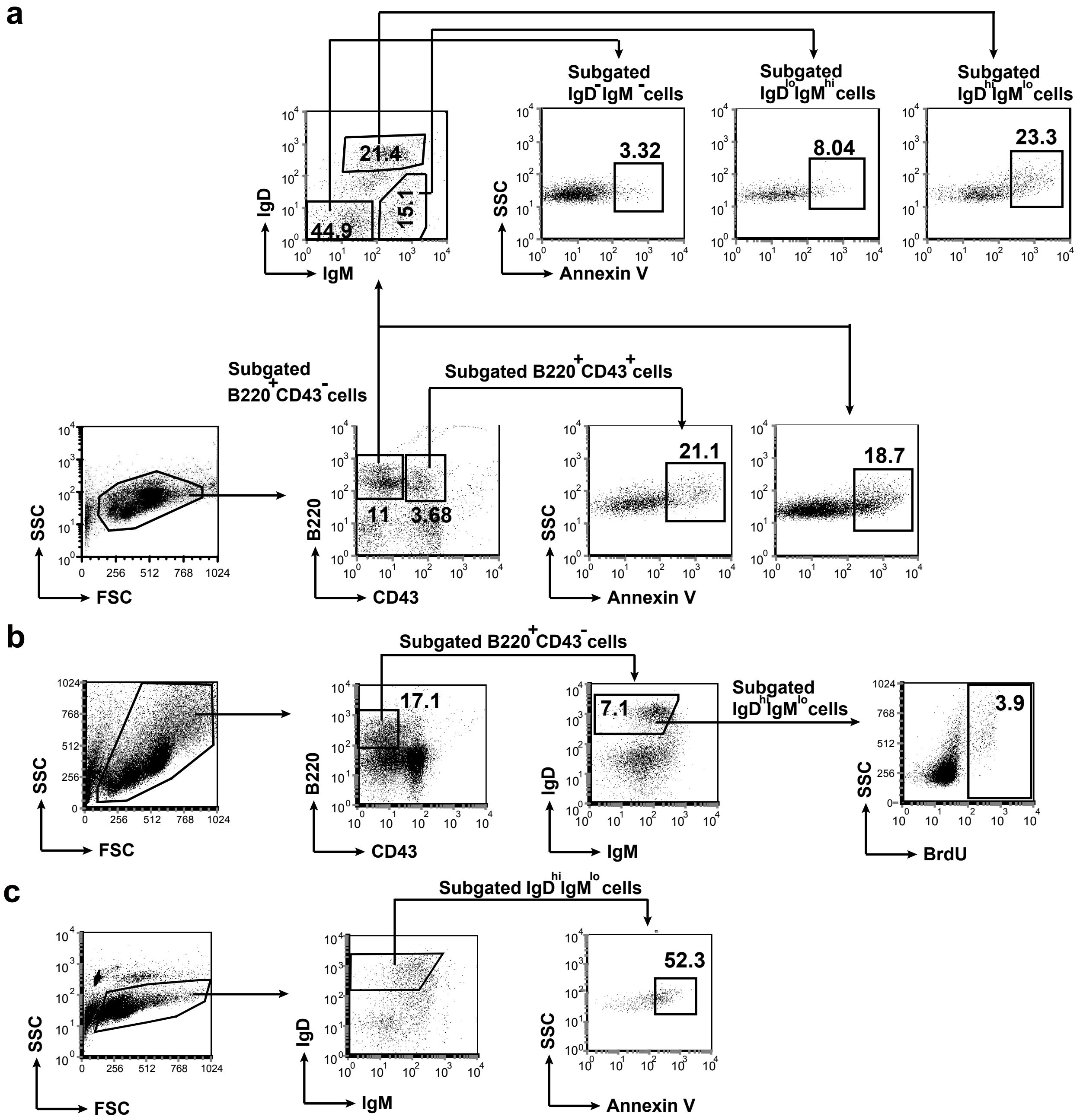

Supplementary Figure 13. Gating strategy for Figure 2

a. Gating strategy for Figures 2a and 2b. Bone marrow cells were first gated by size and granularity (FSC and SSC), and then by B220 and CD43 expression ($B220^{+}CD43^{+}$ GL pro-B, pro-B and early pre-B cells; $B220^{+}CD43^{-}$: late pre-B, immature B and mature B cells). Annexin V staining data for each subpopulation is shown in Figure 2a. $B220^{+}CD43^{-}$ cells were further gated by IgM and IgD expression for late pre-B ($IgM^{-}IgD^{-}$), immature B ($IgM^{hi}IgD^{lo\text{-}int}$) and mature B ($IgM^{int\text{-}hi}IgD^{hi}$) cells. Annexin V staining data for each subpopulation is shown in Figure 2b.

b. Gating strategy for Figure 2c. Bone marrow cells from BrdU-treated mice were first gated by size and granularity (FSC and SSC), and then by B220 and CD43 expression for $B220^{+}CD43^{-}$ cells (including late pre-B, immature B and mature B cells). $B220^{+}CD43^{-}$ cells were further gated by IgM and IgD expression for mature B ($IgM^{int\text{-}hi}IgD^{hi}$) cells. BrdU nuclear incorporation in mature B cells, as visualized by anti-BrdU antibody, is shown in Figure 2c.

c. Gating strategy for Figure 2d. Bone marrow B cells, cultured in vitro for 72 h in the presence or absence of BAFF, were first gated by size and granularity (FSC and SSC), and then by IgM and IgD expression for mature B ($IgM^{int\text{-}hi}IgD^{hi}$) cells. Annexin V staining data for mature B cells is shown in Figure 2d.

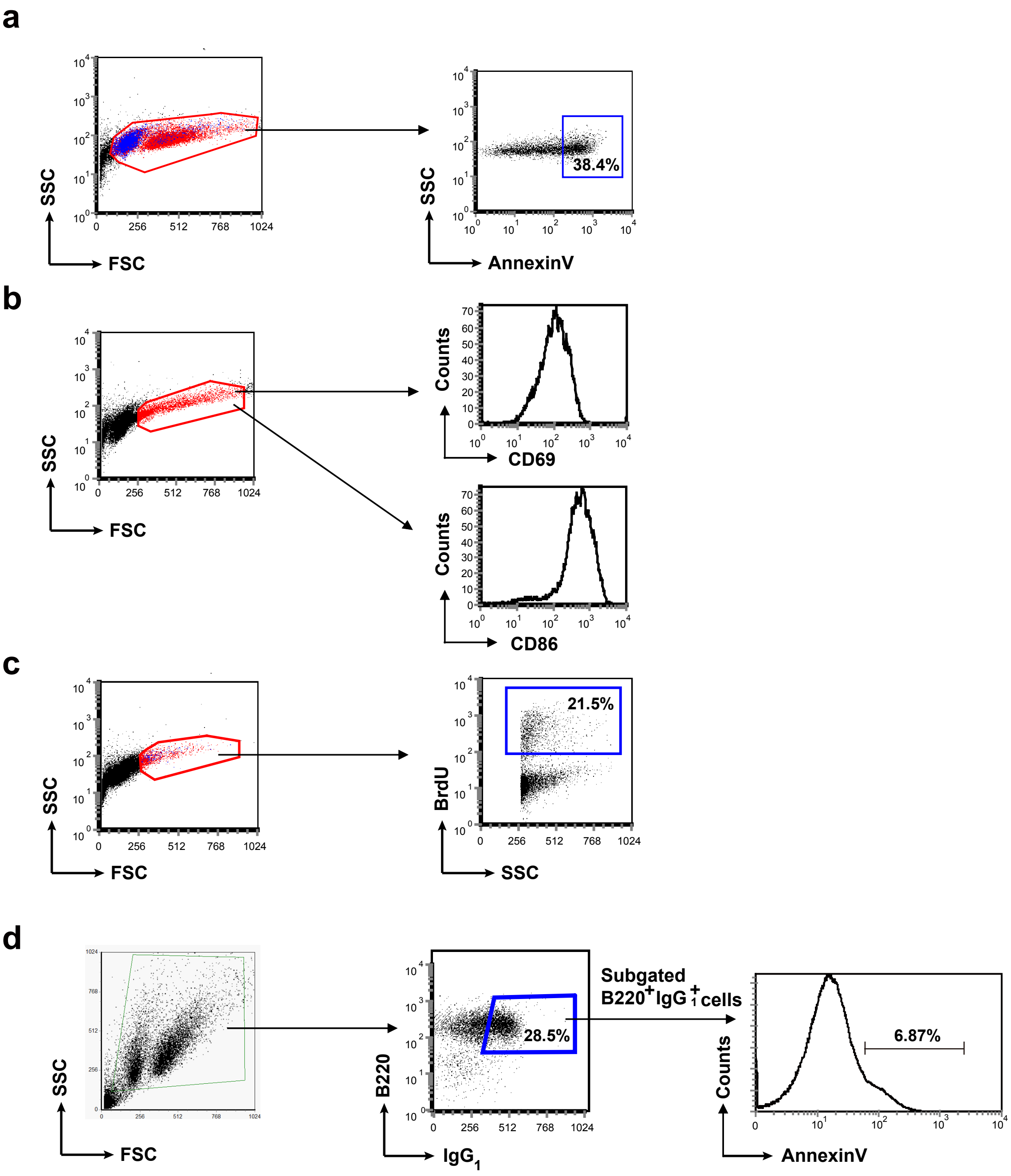

Supplementary Figure 14. Gating strategy for Figure 3 and Figure 5

a. Gating strategy for Figure 3a. Splenic B cells, cultured in vitro in the presence of anti-IgM or LPS for 24 h, were gated by size and granularity (FSC and SSC). Annexin V staining data for the intact cells is shown in Figure 3a.
b. Gating strategy for Figure 3e. Splenic B cells, cultured *in vitro* in the presence of anti-IgM or LPS for 24 h, were gated by size and granularity (FSC and SSC). CD69 and CD86 staining data for the intact cells are shown in Figure 3e.
c. Gating strategy for Figure 3f. Splenic B cells, cultured in vitro in the presence of anti-IgM or LPS for 24 h, were gated by size and granularity (FSC and SSC). BrdU nuclear incorporation in the cells, as visualized by anti-BrdU antibody, is shown in Figure 3e.
d. Gating strategy for Figure 5a. Splenic B cells, stimulated with anti-IgM, anti-CD40 and IL-21 for 2 days, were gated by size and granularity (FSC and SSC) and then with B220 and IgG1 expression. Annexin V staining of $B220^{+}IgG1^{+}$ cells is shown in Figure 5a.

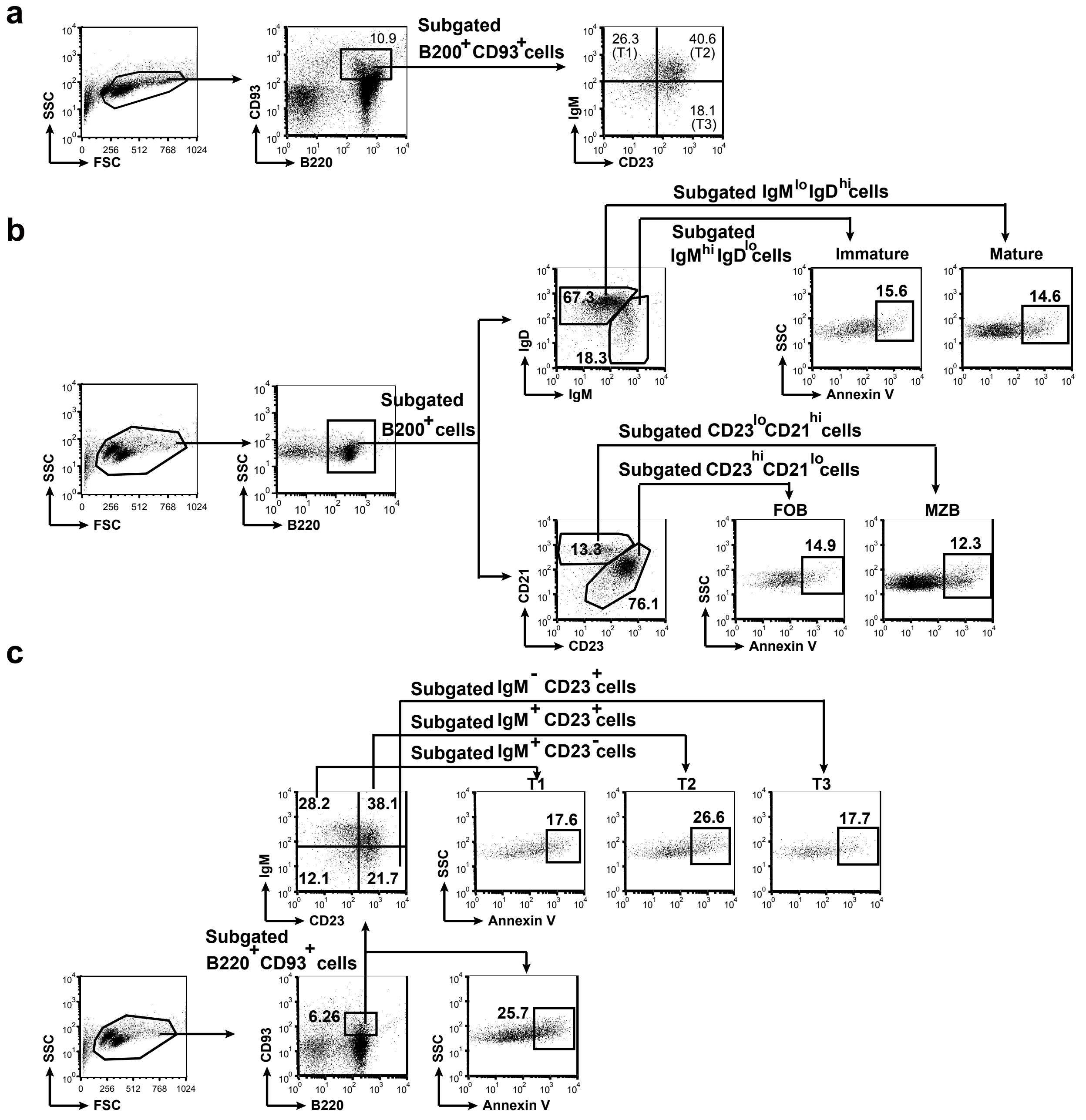

Supplementary Figure 15. Gating strategy for Supplementary Figure 2

a. Gating strategy for Supplementary Figure 2a. Splenic cells were first gated by size and granularity (FSC and SSC), and then by B220 and CD93. $B220^{+}CD93^{+}$ transitional B cells were further gated by CD23 and IgM expression for T1 ($IgM^{+}CD23^{-}$), T2 ($IgM^{+}CD23^{+}$) and T3 ($IgM^{-}CD23^{+}$) cells.

b. Gating strategy for Supplementary Figures 2c and 2d. Splenic cells were first gated by size and granularity (FSC and SSC), and then by B220 expression. B cells ($B220^{+}$) were further gated by IgM and IgD expression for immature ($IgM^{hi}IgD^{lo}$) or mature ($IgM^{lo}IgD^{hi}$) B cells. Annexin V staining data for each subpopulation is shown in Supplementary Figure 2c. Alternatively, B cells ($B220^{+}$) were further gated by CD23 and CD21 expression for MZB ($CD21^{hi}CD23^{lo}$) or FOB ($CD21^{lo}CD23^{hi}$) cells. Annexin V staining data for each subpopulation is shown in Supplementary Figure 2d.

c. Gating strategy for Supplementary Figures 2e and 2f. Splenic cells were first gated by size and granularity (FSC and SSC), and then by B220 and CD93. Annexin V staining data for transitional B cells ($B220^{+}CD93^{+}$) is shown in Supplementary Figure 2e. $B220^{+}CD93^{+}$ transitional B cells were further gated by CD23 and IgM expression for T1 ($IgM^{+}CD23^{-}$), T2 ($IgM^{+}CD23^{+}$) and T3 ($IgM^{-}CD23^{+}$) cells. Annexin V staining data for each subpopulation is shown in Supplementary Figure 2f.
