# Supplementary material for: O-GlcNAcylation is required for B cell homeostasis and antibody responses
Source: Nat Commun. 2017 Nov 30;8:1854. doi: 10.1038/s41467-017-01677-z (PMC5707376; doi:10.1038/s41467-017-01677-z)
Supplement: Supplementary file 3 — Description of Additional Supplementary Files [file 41467_2017_1677_MOESM3_ESM.pdf]

Description of Additional Supplementary Files

File Name: Supplementary Data 1
Description: MS analysis of sWGA pulled-down proteins from Ctrl and B-KO splenic B cells
